# Atypical Hemolytic Uremic Syndrome in Children and Adults With the Hot Spot C3 Gene Variant p.Arg161Trp

**DOI:** 10.1016/j.ekir.2026.106599

**Published:** 2026-05-16

**Authors:** Lieke ter Steeg, Romy N. Bouwmeester, Mendy ter Avest, Frederike J. Bemelman, Antonia H.M. Bouts, Obbo W. Bredewold, Eiske Dorresteijn, Mark Eijgelsheim, Flore A.P.T. Engels, Valentina Gracchi, Rob ter Heine, Mandy G. Keijzer-Veen, Anne-Els van de Logt, Wilbert A.G. van der Meijden, Roos W.G. van Rooij, David Severs, Sjoerd A.M.E.G. Timmermans, Arjan D. van Zuilen, Marloes A.H.M. Michels, Kioa L. Wijnsma, Lambertus P.W.J. van den Heuvel, Jack F.M. Wetzels, Caroline Duineveld, Nicole C.A.J. van de Kar

**Affiliations:** 1 Department of Pediatric Nephrology, Amalia Children’s Hospital, Radboud University Medical Center, Nijmegen, The Netherlands; 2 Department of Pharmacy, Pharmacology & Toxicology, Radboud University Medical Center, Nijmegen, The Netherlands; 3 Department of Nephrology, Amsterdam University Medical Center, Amsterdam, The Netherlands; 4 Department of Pediatric Nephrology, Emma Children’s Hospital, Amsterdam University Medical Center, Amsterdam, The Netherlands; 5 Department of Nephrology, Leiden University Medical Center, Leiden, The Netherlands; 6 Department of Pediatric Nephrology, Sophia Children’s Hospital, Erasmus Medical Center, Rotterdam, The Netherlands; 7 Department of Internal Medicine, Division of Nephrology, University Medical Center Groningen, University of Groningen, Groningen, The Netherlands; 8 Department of Pediatric Nephrology, MosaKids Children’s Hospital, Maastricht University Medical Center, Maastricht, The Netherlands; 9 Department of Pediatric Nephrology, Beatrix Children’s Hospital, University Medical Center Groningen, University of Groningen, Groningen, The Netherlands; 10 Department of Pediatric Nephrology, Wilhelmina Children’s Hospital, University Medical Center Utrecht, Utrecht, The Netherlands; 11 Department of Nephrology, Radboud University Medical Center, Nijmegen, The Netherlands; 12 Department of Pediatric Nephrology, Willem-Alexander Children’s Hospital, Leiden University Medical Center, Leiden, The Netherlands; 13 Department of Internal Medicine, Division of Nephrology and Transplantation, Erasmus Medical Center, University Medical Center Rotterdam, Rotterdam, The Netherlands; 14 Department of Internal Medicine, Section Nephrology and Immunology, Maastricht University Medical Center, Maastricht, The Netherlands; 15 Department of Nephrology and Hypertension, University Medical Center Utrecht, Utrecht, The Netherlands; 16 Department of Genetics, Translational Metabolic Laboratory, Radboud University Medical Center, Nijmegen, The Netherlands; 17 Department of Pediatrics/Pediatric Nephrology, Department of Development and Regeneration Leuven, University Hospitals Leuven, Leuven, Belgium

**Keywords:** atypical hemolytic uremic syndrome, C3 p.Arg161Trp, clinical phenotype, complement, eculizumab, thrombotic microangiopathy

## Abstract

**Introduction:**

The gain-of-function variant C3 p.Arg161Trp is found in a quarter of Dutch patients with complement-mediated atypical hemolytic uremic syndrome (CaHUS). In this study, we describe the clinical phenotype of C3 p.Arg161Trp–associated CaHUS.

**Methods:**

Dutch patients with CaHUS with the C3 p.Arg161Trp variant, identified before October 2023, were included in this retrospective, observational study.

**Results:**

A total of 37 patients (11 children and 26 adults) were included, with onset before and after availability of eculizumab. Presentation at onset differed between children and adults, specifically in platelet count (21 vs. 79 x 10^9^/L, *P* = 0.036), lactate dehydrogenase (2644 vs. 1260 U/L, *P* = 0.026), estimated glomerular filtration rate (eGFR) (48 vs. 15 ml/min per 1.73 m^2^, *P* < 0.001), red-colored urine (90 vs. 24%, *P* = 0.001), and jaundice (50 vs. 10%, *P* = 0.022). None of the children developed end-stage kidney disease (ESKD) within a year after disease onset, in contrast to 11 of 26 adults. Ten children initially showed full recovery, of which 1 had received eculizumab. In adults, kidney function recovery occurred more often in those treated with eculizumab (88%, *n* = 7/8), compared with adults not treated with eculizumab (44%, *n* = 8/18). Relapse rate in the native kidneys of children and adults was high, 82% and 86%, respectively. The median (range) time between disease onset and relapse was 1.9 (0.3–17.6) years.

**Conclusion:**

The clinical phenotype of C3 p.Arg161Trp–associated CaHUS has significant heterogeneity, with differences in presentation and outcomes between children and adults. Children present with more profound hemolysis but milder acute kidney injury (AKI). Furthermore, C3 p.Arg161Trp is linked to a high risk of relapse.

## Graphical abstract

**Figure:**
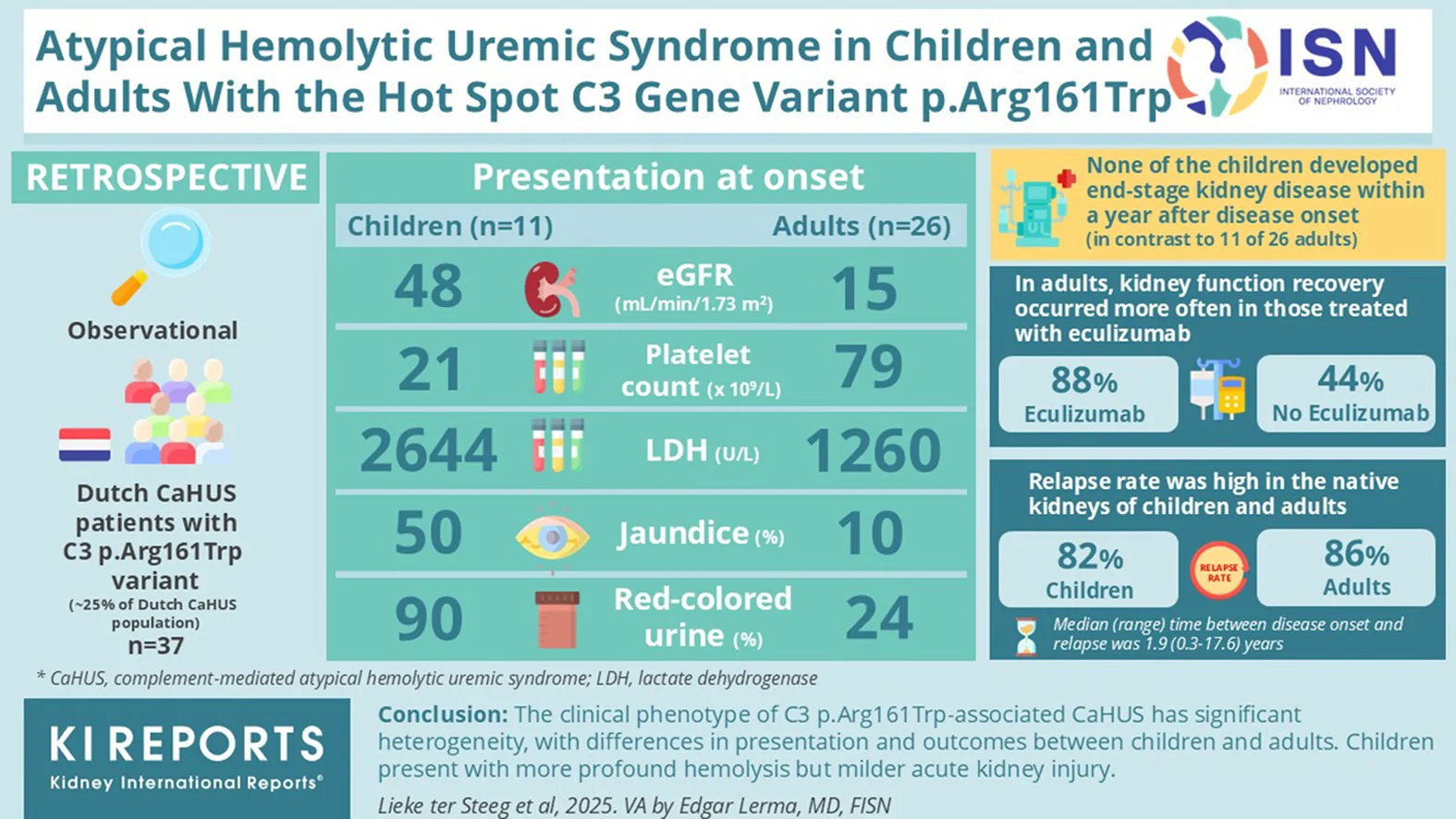

CaHUS is a rare form of thrombotic microangiopathy (TMA) characterized by microvascular thrombosis in the kidneys due to complement dysregulation, resulting in hemolytic anemia, thrombocytopenia, and AKI.^1^ Complement dysregulation is often driven by pathogenic variants in complement (regulatory) proteins, including gain-of-function variants in C3.^2^^,^^3^ Incomplete penetrance has been reported for all pathogenic variants in CaHUS, and is influenced by environmental triggers and genetic predisposition, like the presence of risk haplotypes.^2^^,^^4^^,^^5^

There is considerable heterogeneity in CaHUS phenotype. Still, a distinct relationship between genetic background and clinical manifestation and outcome has been reported.^2^^,^^6^ Patients with pathogenic variants in complement factor H (*CFH*) have a worse prognosis characterized by a high risk of ESKD (∼ 80% after 5 years when not treated with eculizumab), whereas patients with pathogenic variants in Membrane Cofactor Protein (*MCP*) have better kidney outcome, but a high risk of disease recurrence.^2^^,^^7^ This knowledge on genotype-phenotype correlations guides individualized assessment of treatment and prognosis. Nevertheless, because CaHUS is an ultrarare disease with an incidence of approximately 0.23 to 1.9 per million population annually,^8^ investigating the genotype-phenotype correlation of CaHUS-associated pathogenic variants is impeded by small patient cohorts.

In a study of 3128 patients with CaHUS from the Netherlands, United Kingdom, France, Italy, Spain, and the United States of America, a heterozygous gain-of-function variant in the *C3* gene (c.481C>T, p.Arg161Trp) was the most frequent rare variant (1.16%).^9^ Remarkably, this variant is found in a quarter of Dutch patients with CaHUS; the CUREiHUS study and Limburg Renal Registry previously reported, respectively, 24% (*n* = 5/21) and 28% (*n* = 8/28) of patients with CaHUS with this variant.^10^^,^^11^ This is considerably higher than other European and non-European CaHUS cohorts, where allele frequencies of C3 p.Arg161Trp ranged from 0.2% to 4%.^7^^,^^9^^,^^12^^,^^13^ This variant is considered pathogenic, because it leads to increased binding of factor Bb to C3b and decreased binding of complement regulators, resulting in increased complement activity.^12^^,^^13^ Localization and functional consequences of C3 p.Arg161Trp are illustrated in Supplementary Figure S1.

The clinical presentation of C3 p.Arg161Trp–associated CaHUS has been described in previous studies; and suggested high frequency of extrarenal manifestations, chronic histologic features of TMA, and poor kidney outcome.^11^^,^^12^ ESKD was seen in 6 of 7 patients and 6 of 14 patients 1 year after disease onset, respectively.^11^^,^^12^ These observations were, however, derived from small patient cohorts treated in the pre-eculizumab era. In this study, we aimed to provide an in-depth analysis of the genotype-phenotype correlation in C3 p.Arg161Trp–associated CaHUS, leveraging the high prevalence of this variant among Dutch patients with CaHUS.

## Methods

In this retrospective, observational study, we evaluated patients with CaHUS with the C3 p.Arg161Trp variant in the Netherlands. Eligibility for inclusion required identification of the heterozygous C3 p.Arg161Trp variant before October 2023, either through previous genetic analysis (requested by a [pediatric] nephrology department) or by documentation in medical records at Radboud University Medical Center. Only patients diagnosed with CaHUS by their treating physician were included. Individuals without available clinical data were excluded. Retrospective data were collected from medical records and encompassed demographics, medical history, clinical and diagnostic findings, treatment, and follow-up data from disease onset up to October 2023. Definitions for chronic kidney disease and (severe) hypertension are described in Supplementary Method S1.

Data were classified by age at onset of CaHUS–pediatric (aged < 18 years) and adult (aged ≥ 18 years). To reassess CaHUS diagnosis, first CaHUS episodes were retrospectively analyzed for the presence of AKI and laboratory and/or histological evidence of TMA (Supplementary Method S2).

### Genetic Analysis

Genetic analysis data were collected for CaHUS-associated genes, as routinely performed in all patients with CaHUS in the Netherlands.^10^ Genetic variants were classified as (likely) benign, variant of uncertain significance, or (likely) pathogenic.^14^

### Outcomes

The study cohort consisted of patients treated before and after eculizumab approval in the Netherlands in 2012. Since 2016, most patients with CaHUS in the Netherlands have been treated according to a restrictive eculizumab protocol,^15^ which involves the cessation or dose interval prolongation of eculizumab after 3 months of treatment. According to treatment protocol, patients without a (familial) history of TMA were first treated with plasma exchange for a 4-day period, providing time for diagnostics into other causes of TMA. If patients did not respond to plasma exchange and no other cause of TMA was found, eculizumab treatment was started.

We evaluated the frequency of relapse. A relapse was either defined as the need for restart or intensification of treatment (eculizumab or plasma exchange) or identified as recurrent CaHUS by the treating physician (in case of supportive treatment). All relapses were retrospectively classified as suspected or definite. Relapses were considered definite in the presence of AKI or acute kidney disease in combination with laboratory evidence of TMA. Patients were not or no longer considered to be at risk for relapse after developing ESKD or while receiving eculizumab.

Kidney recovery was assessed 1 year after CaHUS onset. In case of a relapse or last follow-up visit < 1 year after onset, recovery was assessed using the last known laboratory values before the relapse or at the time of last follow-up visit. Full recovery was defined as a recovery of kidney function to normal serum creatinine level (corrected for age and sex) and absence of proteinuria (< 0.10 g/l or <10 mg/mmol creatinine in adults, <23 mg/mmol creatinine in children). Partial recovery was defined as a recovery of kidney function but with persistently increased serum creatinine level (corrected for age and sex) and/or presence of proteinuria. ESKD was defined as the need for persistent dialysis and/or kidney transplantation. eGFR was calculated using the Chronic Kidney Disease–Epidemiology Collaboration formula in adults and the revised bedside Schwartz formula in children.^16^^,^^17^ Kidney outcomes were assessed for all patients after relapse and at last follow-up.

### Statistical Analysis

Continuous variables were reported as median and range (minimum–maximum). Groups were compared using the Mann-Whitney U test or Kruskall-Wallis test for nonnormally distributed data. Categorical variables were reported as absolute numbers and percentages, and compared using the chi-square test or Fisher exact test; for comparisons involving multiple groups, a Monte Carlo analysis was applied. A *P* value < 0.05 was considered significant. No correction was made for multiple testing, apart from pairwise comparisons following a Kruskall-Wallis test. Instead, individual *P*-values were noted. Kaplan-Meier analysis was used to examine kidney survival. Statistical analyses were performed using SPSS software package (version 29.0, IBM Corp.), and figures were drawn using Biorender (Biorender.com) and Microsoft Excel (version 365, Microsoft Corp.).

### Ethics

A subset of patients with CaHUS were enrolled in the CUREiHUS study, a nationwide multicenter observational study that monitored patients with CaHUS treated with a restrictive eculizumab protocol.^10^^,^^18^ The patients provided written informed consent. Additional patients with CaHUS were recruited using passive (opt out) informed consent for inclusion in a national TMA database. Ethical approval for both studies was granted by the Medical Research Ethics Committee of Oost-Nederland (CUREiHUS: NL52817, and TMA database: 2019-5676). Of note, 7 patients with CaHUS were included in previous reports^10^^,^^18^ and are presented here with extended follow-up.

## Results

**Figure 1:**
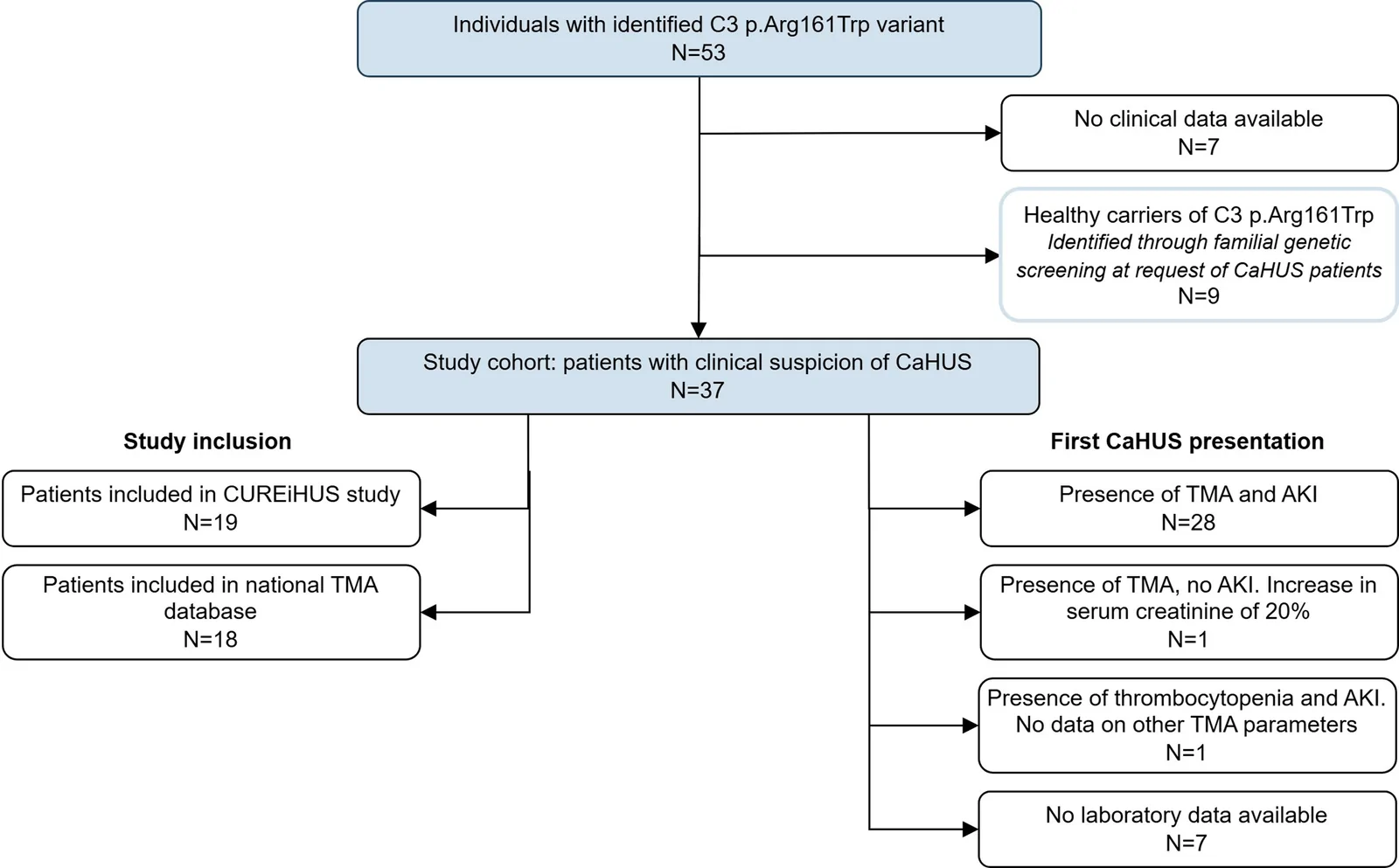
Flowchart of study cohort selection and characterization of CaHUS onset. AKI, acute kidney injury; CaHUS, complement-mediated atypical hemolytic uremic syndrome; TMA, thrombotic microangiopathy.

In total, 53 individuals with the heterozygous C3 p.Arg161Trp variant were identified, of whom 37 patients with a clinical suspicion of CaHUS were included in this study (Figure 1). Twenty-one patients presented before availability of eculizumab in the Netherlands in 2012. Median age at CaHUS onset was 32 years, and there were 11 children and 26 adults (Table 1).

**Table 1:** Characteristics of patients with CaHUS with the heterozygous C3 p.Arg161Trp variant

Characteristics			Total^a^ *N* = 37		Children (C) *n* = 11		Adults (A) *n* = 26	*P* value C vs. A
Median age at presentation (range), yr			32 (0-62)		6 (0-16)		38 (18-62)	-
Sex								-
Female			19 (51%)		5 (45%)		14 (54%)	*P* = 0.728
Male			18 (49%)		6 (55%)		12 (46%)	
Medical history^b^								
Cardiovascular and/or kidney disease		*n =* 37	13 (35%)	*n =* 11	0	*n =* 26	13 (50%)	*P* = 0.003
Hypertension		*n =* 37	8 (22%)	*n =* 11	0	*n =* 26	8 (31%)	-
CKD		*n =* 37	6 (16%)	*n =* 11	0	*n =* 26	6 (23%)	-
Hypertensive disorders of pregnancy		*n =* 37	4 (11%)	*n =* 11	0	*n =* 26	4 (15%)	-
Concomitant autoimmune and/or kidney disease^c^		*n =* 37	2 (8%)	*n =* 11	0	*n =* 26	2 (8%)	*P* = 1.000
Family history^d^								
Familial CaHUS		*n =* 37	18 (49%)	*n =* 11	7 (64%)	*n =* 26	11 (42%)	*P* = 0.295
Unspecified kidney disease		*n =* 37	2 (5%)	*n =* 11	0	*n =* 26	2 (8%)	-
Genetic analysis								
Patients with additional complement genetic variant(s)^e^		*n =* 37	3 (8%)	*n =* 11	1 (9%)	*n =* 26	2 (8%)	*P* = 1.000
MCP_ggaac_ haplotype homozygosity		*n =* 36	22 (61%)	*n =* 11	9 (82%)	*n =* 25	13 (52%)	*P* = 0.142
CFH-H3 haplotype homozygosity		*n =* 37	3 (8%)	*n =* 11	1 (9%)	*n =* 26	2 (8%)	*P* = 1.000
Symptoms at presentation^f^								
No symptoms		*n =* 31	0	*n =* 10	0	*n =* 21	0	
Petechiae		*n =* 31	6 (19%)	*n =* 10	4 (40%)	*n =* 21	2 (10%)	*P* = 0.067
Jaundice		*n =* 31	7 (23%)	*n =* 10	5 (50%)	*n =* 21	2 (10%)	*P* = 0.022
Red-colored urine		*n =* 31	14 (45%)	*n =* 10	9 (90%)	*n =* 21	5 (24%)	*P* = 0.001
Hypertension at presentation								
Hypertension		*n =* 29	28 (97%)	*n =* 8	7 (88%)	*n =* 21	21 (100%)	*P* = 0.276
Severe hypertension		*n =* 29	16 (55%)	*n =* 8	3 (38%)	*n =* 21	13 (62%)	*P* = 0.406
Extrarenal manifestations at presentation								
Extrarenal manifestations		*n =* 37	7 (19%)	*n =* 11	1 (9%)	*n =* 26	6 (23%)	*P* = 0.649
Neurological		*n =* 37	2 (5%)	*n =* 11	1 (9%)	*n =* 26	1 (4%)	-
Cardiac		*n =* 37	3 (8%)	*n =* 11	0	*n =* 26	3 (12%)	-
Respiratory		*n =* 37	3 (8%)	*n =* 11	0	*n =* 26	3 (12%)	-
Other		*n =* 37	0	*n =* 11	0	*n =* 26	0	-
Triggers								
Viral like symptoms		*n =* 37	19 (51%)	*n =* 11	8 (73%)	*n =* 26	11 (42%)	*P* = 0.151
Proven viral infection	Well-defined triggers	*n =* 37	3 (8%)^g^	*n =* 11	2 (18%)	*n =* 26	1 (4%)^g^	*P* = 0.205
Bacterial infection	Well-defined triggers	*n =* 37	3 (8%)	*n =* 11	0	*n =* 26	3 (12%)	*P* = 0.540
Respiratory infection	Well-defined triggers	*n =* 37	0	*n =* 11	0	*n =* 26	0	-
Gastrointestinal infection	Well-defined triggers	*n =* 37	1 (3%)^g^	*n =* 11	0	*n =* 26	1 (4%)^g^	-
Other infection	Well-defined triggers	*n =* 37	2 (5%)	*n =* 11	0	*n =* 26	2 (8%)	-
Vaccination	Well-defined triggers	*n =* 37	1 (3%)	*n =* 11	1 (9%)	*n =* 26	0	*P* = 0.297
Pregnancy	Well-defined triggers	*n =* 37	2 (5%)	*n =* 11	0	*n =* 26	2 (8%)	*P* = 1.000
Medication	Well-defined triggers	*n =* 37	1 (3%)^h^	*n =* 11	1 (9%)^h^	*n =* 26	0	*P* = 0.297
No reported trigger		*n =* 37	13 (35%)	*n =* 11	2 (18%)	*n =* 26	11 (42%)	*P* = 0.262

CaHUS, complement-mediated atypical hemolytic uremic syndrome; CFH, complement factor H; CKD, chronic kidney disease; MCP, membrane cofactor protein.

a *n* refers to the number of patients for whom data were available.

b Medical history included 8 patients with hypertension, 6 patients with chronic kidney disease, 2 patients with familial hypercholesterolemia, 1 patient with myocardial infarction, and 4 patients with hypertensive disorders of pregnancy (one patient with Hemolysis, Elevated Liver enzyme levels, and Low Platelet Levels, 1 patient with gestational hypertension, and 2 patients with preeclampsia).

c One patient was diagnosed with antiphospholipid syndrome and systemic lupus erythematosus. One patient was diagnosed with antiphospholipid syndrome.

d Familial CaHUS was defined as the presence of CaHUS in ≥1 family member. Unaffected family members with the C3 p.Arg161Trp variant were not included. Family members with unspecified kidney disease were also not included but separately noted.

e Including variants in complement genes classified as class III variants (unknown significance), class IV variants (likely pathogenic), and class V variants (pathogenic).

f Only symptoms with different frequency between children and adults are given in the table. A complete list of symptoms is included in Supplementary Table S2 in the appendix.

g This patient suffered from a COVID-19 infection 1 month prior to CaHUS, and a STEC infection 1 week before CaHUS (both PCR positive and clinical signs of STEC, no bloody diarrhea).

h This patient experienced CaHUS onset shortly after start of an oral contraceptive. No other trigger was identified. Median values (ranges) are shown. Percentages may not total 100% due to rounding.

### Genetic and Clinical Background

In addition to the C3 p.Arg161Trp variant, 3 patients carried another (likely) pathogenic variant or a variant of unknown significance in CaHUS-associated genes (Supplementary Table S1). The *MCP*_ggaac_ and *CFH*-H3 risk haplotype were present in homozygosity in respectively 22 of 36 (61%) and 3 of 37 (8%) patients.

A history of cardiovascular and/or kidney disease was reported in 50% of adults, but in none of the children. One adult had previously been diagnosed with membranous nephropathy. Following the onset of TMA, concomitant autoimmune and/or kidney disease were identified in 2 adults: both had antiphospholipid syndrome, and 1 had systemic lupus erythematosus but without lupus nephritis.

### Clinical Characteristics at Onset

All the patients experienced CaHUS onset in their native kidneys. Among those with available laboratory data at onset (*n* = 30), all but 2 patients exhibited TMA and AKI (Figure 1). A well-defined trigger for CaHUS was identified in 9 patients (24%), most commonly, laboratory-confirmed infection (*n* = 5, 14%) and pregnancy (*n* = 2, 5%). Severe hypertension was present at onset in 16 patients (55%).

**Figure 2:**
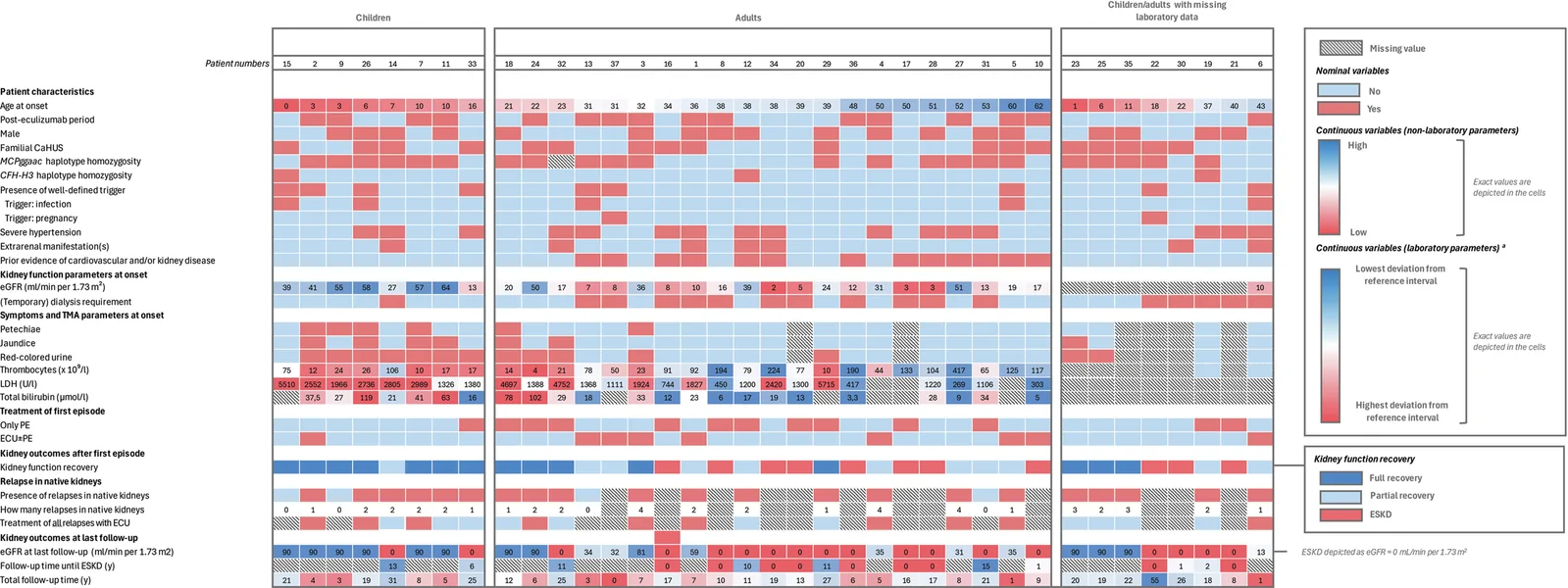
Heatmap illustrating clinical heterogeneity of pediatric and adult patients with the C3 p.Arg161Trp variant. The pattern of hematological TMA and kidney function parameters shifts across age groups, with older patients showing less prominent hematological TMA and worse kidney function. Children generally had favorable kidney outcomes after their first presentation and at last follow-up. In adults, those treated with eculizumab—both at the first presentation and during relapses—demonstrated better kidney outcomes compared with adults who did not receive eculizumab. ^a^For each laboratory value, the relative deviation from the reference interval was calculated, with values within the interval assigned zero. Deviations were normalized by scaling to the maximum observed deviation for each laboratory value. Because deviations for LDH and total bilirubin showed a highly skewed distribution, normalization for these parameters was capped at predefined thresholds (> 2000 IU/l for LDH and > 51 μmol/l for total bilirubin). The color scale reflects the relative magnitude of deviation across the dataset, ranging from smaller (blue) to larger (red) deviations, without encoding the direction of deviation (i.e. above or below the reference interval). CFH, complement factor H; ECU, eculizumab; eGFR, estimated glomerular filtration rate; ESKD, end-stage kidney disease; LDH, lactate dehydrogenase; MCP, membrane cofactor protein; PE, plasma exchange; TMA, thrombotic microangiopathy.

Differences in symptoms and laboratory findings were observed between children and adults (Table 1 and Supplementary Table S2). Red-colored urine (90 vs. 24%, *P* = 0.001) and jaundice (50 vs. 10%, *P* = 0.022) were reported more often in children than in adults, parallelled by significantly lower platelet counts (21 vs. 79 x 10^9^/L, *P* = 0.036), and higher levels of lactate dehydrogenase (2644 vs. 1260 U/l, *P* = 0.026), total bilirubin (38 vs. 19 μmol/l, *P* = 0.076), and aspartate aminotransferase (115 vs. 70 U/l, *P* = 0.078) in children than in adults (Table 2). Renal impairment was less severe in children, as evidenced by a higher eGFR (48 vs. 15 ml/min per 1.73 m^2^, *P* < 0.001) and a reduced need for acute dialysis (9 vs. 62%, *P* = 0.004). A heatmap displaying the clinical features of all patients similarly highlights distinct patterns of presentation with more severe signs of hematological TMA and less severe kidney injury at a younger age (Figure 2).

**Table 2:** Laboratory findings at presentation of patients with CaHUS with the C3 p.Arg161Trp variant

Characteristics		Total^a^ *N* = 37		Children (C) *n* = 11		Adults (A) *n* = 26	*P-*value C vs. A
Serum creatinine (μmol/l)	*n* = 30	303 (61–1730)	*n =* 8	85 (61–453)	*n =* 22	369 (108–1730)	-
eGFR (ml/min per 1.73 m^2^)	*n* = 30	18 (2–64)	*n =* 8	48 (13–64)	*n =* 22	15 (2–51)	*P* < 0.001
Thrombocytes (x 10^9^/l)	*n* = 29	75 (4–417)	*n =* 8	21 (10–106)	*n =* 21	79 (4–417)	*P* = 0.036
Haptoglobin < 0.3 g/l (*n* (%))	*n* = 21	20 (95%)	*n =* 6	5 (83%)	*n =* 15	15 (100%)	*P* = 0.286
LDH (U/l)	*n* = 26	1384 (269–5715)	*n =* 8	2644 (1326–5510)	*n =* 18	1260 (269–5715)	*P* = 0.026
Hemoglobin (mmol/l)	*n* = 30	5.9 (3.6–8.6)	*n =* 8	6.0 (3.6–6.6)	*n =* 22	5.7 (4.1–8.6)	*P* = 0.447
UPCR (g/l)	*n* = 14	4.55 (0.63–9.80)	*n =* 2	6.44 (6.40–6.47)	*n =* 12	4.05 (0.63–9.80)	*P* = 0.352
ASAT (U/l)	*n* = 17	84 (23–312)	*n =* 6	115 (65–160)	*n =* 11	70 (23–312)	*P* = 0.078
Total bilirubin (μmol/l)	*n* = 23	23 (3–119)	*n =* 7	38 (16–119)	*n =* 16	19 (3–102)	*P* = 0.076

ASAT, aspartate aminotransferase; eGFR, estimated glomerular filtration rate; LDH, lactate dehydrogenase; UPCR, urine protein-to-creatinine ratio.

a *n* refers to the number of patients for whom data were available. Median values (ranges) are shown.

### Treatment and Outcomes

In view of the differences in presentation between children and adults, treatment and outcomes were described separately for each group. In all children, kidney function recovered after the first presentation, with 10 achieving full recovery and 1 showing partial recovery (Figure 2). Notably, 9 of 10 children achieving full recovery were treated supportively (no plasma exchange or eculizumab). In these children, spontaneous recovery of TMA and kidney function occurred within a few days after presentation (data not shown). One child received eculizumab, which was discontinued after 7.1 months. The only child with partial recovery (P14) was treated supportively, presented with a lower eGFR necessitating temporary dialysis, and had a higher platelet count.

In adults, kidney function recovery after the first presentation was observed in only 15 of 26 patients (58%), with partial recovery in 10 and full recovery in 5. Overall, 8 of 26 adults were treated with eculizumab, with recovery of kidney function in 7, whereas recovery was observed in only 8 of 18 adults not treated with eculizumab (88% vs. 44%, *P* = 0.084). The patient (P10) who developed ESKD after receiving eculizumab had a substantial delay in start of eculizumab treatment (Supplementary Result S1). Kidney function recovery outcomes, stratified by presentation in the pre- and post-eculizumab eras, are detailed in Supplementary Table S3. Of note, the median duration of eculizumab treatment in adults was 3.3 (1.4–6.4) months, and eculizumab was discontinued in all but 1 patient after onset.

Comparison of adult groups with different kidney outcomes (Table 3) shows that adults with full recovery after onset were typically younger, with a median age of 23 (21–39) years, had no prior history of cardiovascular and/or kidney disease, presented with significantly higher eGFR, did not require temporary dialysis, and showed more severe thrombocytopenia and hemolysis, resembling the presentation seen in children. Notably, 2 young adults who progressed to ESKD after the first presentation (P22 and P30) had missing data on hematological TMA and kidney function parameters.

**Table 3:** Laboratory findings and treatment in adults at presentation stratified by kidney outcome after first episode

Characteristics		Adults with full recovery^a^*n* = 5		Adults with partial recovery^b^*n* = 10		Adults with ESKD *n* = 11	*P-*value
Median age at presentation (range), yr		23 (21-39)		40.5 (31-60)		39 (18-62)	*P* = 0.086
Sex							*P* = 0.313
Female		2 (40%)		4 (40%)		8 (73%)	
Male		3 (60%)		6 (60%)		3 (27%)	
Time of presentation							*P* = 0.178
< 2012 (eculizumab not available)		3 (60%)		3 (30%)		8 (73%)	
≥ 2012 (eculizumab available)		2 (40%)		7 (70%)		3 (27%)	
Medical history							
Cardiovascular and/or kidney disease	*n =* 5	0	*n =* 10	7 (70%)	*n =* 11	6 (55%)	*P* = 0.039
Hypertension	*n =* 5	0	*n =* 10	3 (30%)	*n =* 11	5 (45%)	-
CKD	*n =* 5	0	*n =* 10	2 (20%)	*n =* 11	4 (36%)	-
Genetic analysis							
Patients with additional complement genetic variant(s)	*n =* 5	1 (20%)	*n =* 10	1 (10%)	*n =* 11	0	*P* = 0.491
MCP_ggaac_ haplotype homozygosity	*n =* 4	4 (100%)	*n =* 10	7 (70%)	*n =* 11	2 (18%)	*P* = 0.004
CFH-H3 haplotype homozygosity	*n =* 5	0	*n =* 10	2 (20%)	*n =* 11	0	*P* = 0.175
Laboratory findings at presentation							
Serum creatinine (μmol/l)	*n =* 5	272 (131–361)	*n =* 9	407 (108–605)	*n =* 8	647.5 (254–1730)	-
eGFR (ml/min per 1.73 m^2^)	*n =* 5	24 (17–50)	*n =* 9	13 (7–51)	*n =* 8	7 (2–17)	*P* = 0.011^c^
Thrombocytes (x 10^9^/l)	*n =* 5	14 (4–23)	*n =* 8	79 (44–417)	*n =* 8	125 (77–224)	*P* = 0.002^d^
Haptoglobin <0.3 g/l (n (%))	*n =* 5	4 (100%)	*n =* 7	7 (100%)	*n =* 5	4 (100%)	-
LDH (U/l)	*n =* 5	4697 (1388–5715)	*n =* 6	1156 (269–1827)	*n =* 7	744 (303–2420)	*P* = 0.014^c^
Hemoglobin (mmol/l)	*n =* 5	6.1 (5.1–8.6)	*n =* 9	5.6 (4.1–8.5)	*n =* 8	5.4 (4.3–6.0)	*P* = 0.243
UPCR (g/l)	*n =* 2	6.75 (3.69–9.80)	*n =* 6	4.62 (1.91–9.63)	*n =* 4	4.05 (0.63–4.70)	*P* = 0.304
ASAT (U/l)	*n =* 4	101 (71–130)	*n =* 4	59 (44–312)	*n =* 3	32 (23–54)	*P* = 0.071
Total bilirubin (μmol/l)	*n =* 4	56 (29–102)	*n =* 5	18 (9–34)	*n =* 7	12 (3–28)	*P* = 0.014^c^
Need for dialysis							
Dialysis during acute episode	*n =* 5	0	*n =* 10	6 (60%)	*n =* 11	10 (91%)	-
Treatment at presentation							
Supportive treatment	*n =* 5	0	*n =* 10	1 (10%)	*n =* 11	5 (45%)	-
PE	*n =* 5	4 (80%)	*n =* 10	3 (30%)	*n =* 11	5 (45%)	-
Eculizumab ≤ 7 days ± PE	*n =* 5	1 (20%)	*n =* 10	3 (30%)	*n =* 11	0	-
Eculizumab > 7 days ± PE	*n =* 5	0	*n =* 10	3 (30%)	*n =* 11	1 (9%)	-

ASAT, aspartate aminotransferase; CFH, complement factor H; CKD, chronic kidney disease; eGFR, estimated glomerular filtration rate; ESKD, end-stage kidney disease; KDIGO, Kidney Disease: Improving Global Outcomes; LDH, lactate dehydrogenase; MCP, membrane cofactor protein; PE, plasma exchange; UPCR, urine protein-to-creatinine ratio.

Median values (ranges) are shown.

Percentages may not total 100% due to rounding.

a *n* refers to the number of patients for whom data were available.

b According to KDIGO guidelines, patients with partial recovery were classified as follows: 2 patients had CKD stage G3a, 4 patients had CKD stage G3b, 1 patient had CKD stage G5, and 3 patients had unspecified CKD.

c Pairwise comparisons showed only a significant difference (*P* < 0.05) between the full recovery and ESKD groups.

d Pairwise comparisons showed a significant difference (*P* < 0.05) between the full recovery and ESKD groups and between the full recovery and partial recovery groups.

### First Relapse

A suspected or definite relapse occurred in 9 of 11 children (82%) (Table 4). The median time between disease onset and first relapse in children was 5.3 (1.0–17.6) years. Similar to the presentation at onset, children presented with severe thrombocytopenia and hemolysis but mild renal impairment at first relapse (data not shown). Three children were treated with eculizumab. Overall, 7 of 9 children recovered fully (increase in serum creatinine < 10% from baseline). At relapse, their kidney function was relatively preserved with a median eGFR of 49 (18–99) ml/min per 1.73 m^2^. Two children (P14 and P33) experienced loss of kidney function due to disease relapse (increase in serum creatinine ≥ 10% from baseline) despite receiving plasma exchange; they relapsed before eculizumab availability. At relapse, these children presented with eGFRs of 26 and 13 ml/min per 1.73 m^2^, respectively.

**Table 4:** Relapse risk in C3 p.Arg161Trp patients with native kidneys

Characteristics		Total^a^ *n* = 25		Children at risk for relapse *n* = 11		Adults at risk for relapse *n* = 14
Number of patients with a relapse		21 (84%)		9 (82%)		12 (86%)
Total number of relapses in native kidneys		44		18		26
Time between disease onset and first relapse (yr)	*n =* 21	1.9 (0.3–17.6)	*n =* 9	5.3 (1.0–17.6)	*n =* 12	1.4 (0.3–11.1)
Time between eculizumab discontinuation and first relapse (yr)	*n =* 6	0.4 (0.1–2.2)	*n =* 1	0.4	*n =* 5	0.5 (0.1–2.2)

Median values (ranges) are shown.

a *n* refers to the number of patients for whom data were available.

There were 14 adult patients at risk for relapse, of whom 12 (86%) had a suspected or definite relapse. Seven adults were treated with eculizumab, and 6 of them (86%) recovered to baseline kidney function before relapse. The patient who did not recover fully had started eculizumab > 7 days after TMA relapse. In contrast, of the 5 adults who were not treated with eculizumab, only 2 (40%) recovered fully. Notably, there was a difference in time to relapse between patients who had received treatment with eculizumab at onset and who had not. The median time between eculizumab discontinuation and relapse was 0.5 (0.1–2.2) years, compared with a median time of 3.4 (0.3–11.1) years between disease onset and relapse in adults who had not received eculizumab.

In this whole cohort, 21 patients experienced a first relapse in native kidneys and 15 patients had multiple relapses, resulting in 44 suspected relapses in total. Of these, 89% (*n* = 17/19; laboratory data unavailable for *n* = 2) of first relapses and 68% (*n* = 15/22, laboratory data unavailable for *n* = 1) of subsequent relapses could be confirmed as definite relapses.

### Long-Term Outcomes

At 10 years after the first presentation, ESKD-free survival was 88% in children, 86% in adults treated with eculizumab, and 31% in adults not treated with eculizumab (Supplementary Figure S2). Over the entire follow-up (median [range]: 19 [3–31] years), 2 of 11 children (P14 and P33) progressed to ESKD due to disease relapse: P33 following the first relapse and P14 following the second relapse. Noteworthy, the other 9 children still had an eGFR > 90 ml/min per 1.73 m^2^ with little to no proteinuria at the end of follow-up. Of these 9 children with a preserved eGFR at the end of follow-up, 7 had experienced a total of 15 relapses, of which 9 were treated with eculizumab.

In adults, 16 of 26 adults had progressed to ESKD by the end of follow-up (median [range]: 10 [0.3–55] years). Eleven developed ESKD within 1 year after first presentation, and 5 adults developed ESKD >1 year after the first presentation. With the exception of 1 patient (P10), none of the adults who progressed to ESKD were treated with eculizumab at onset or at relapse(s) (Figure 2).

The 10 adults who did not progress to ESKD experienced 19 relapses in total, all but 1 treated with eculizumab. One relapse resulted in additional kidney function loss despite eculizumab treatment, in the other patients relapses did not adversely affect kidney function (Supplementary Figure S3). This patient received eculizumab 14 days after relapse presentation. After 2 dosages, eculizumab treatment was discontinued because of presentation with colitis, initially suspected to be eculizumab-related. Kidney function declined from 48 to 32 ml/min per 1.73 m^2^.

One patient, who had received a kidney transplant after developing ESKD, died during follow-up due to urinary tract–associated sepsis. No deaths due to CaHUS were recorded.

## Discussion

In this study, we describe the clinical phenotype of a large cohort of patients with CaHUS carrying the C3 p.Arg161Trp variant. This variant accounts for a quarter of complement-associated pathogenic variants found in Dutch patients with CaHUS. Our data highlight the clinical heterogeneity in C3 p.Arg161Trp patients, with distinct patterns of presentation and recovery in children versus adults.

Children, compared with adults, presented with more severe hemolysis (with higher lactate dehydrogenase and bilirubin) and thrombocytopenia, but presented with milder AKI. This appears not to be unique to the C3 p.Arg161Trp variant, because similar differences were observed in other CaHUS cohorts when comparing pediatric and adult patients.^10^^,^^19^^,^^20^ Interestingly, signs of hemolysis, such as jaundice and red-colored urine, were observed in 90% of the children with the C3 p.Arg161Trp variant but are uncommon and not systematically reported in patients with CaHUS with pathogenic variants in other complement genes.^7^^,^^21^, ^22^, ^23^ At present, we have no explanation for this finding.

Kidney outcomes after the first presentation were better in children than in adults (0% vs. 42% ESKD), which is also observed in other CaHUS cohorts and independent of the responsible genetic variant.^7^^,^^20^^,^^24^ After the first presentation, full recovery occurred in 91% (*n* = 10/11) of children and in only 19% (*n* = 5/26) of adults. Adults with full recovery resembled pediatric presentation: they tended to be younger (median [range] age: 23 [21–39] years), had no history of cardiovascular or kidney disease, and presented with more severe hemolysis but milder AKI. Notably, only 2 of 15 patients with full recovery had received eculizumab. Previous studies associated C3 p.Arg161Trp with poor kidney outcome,^11^^,^^12^ in contrast to the favorable outcomes observed in the children in our cohort. The cohorts in these previous studies consisted mainly of adults who were treated before availability of eculizumab.

The good kidney outcome in children mirrors to the more preserved eGFR at first presentation (median [range]: 55 [13–64] ml/min per 1.73 m^2^). This may be explained by an earlier diagnosis or higher renal functional reserve in children.^25^ In contrast, few adults showed full recovery. Recovery of kidney function was observed more often in adults treated with eculizumab (88 vs. 44%). Remarkably, these adults had presented with severely decreased eGFR and often needed temporary dialysis. Importantly, half of adult patients had a prior history of cardiovascular or kidney disease. This raises the question of whether these adults may have already developed vascular damage due to subclinical complement-mediated injury before presenting with a typical CaHUS episode. Whatever the cause, existent vascular damage may explain the suboptimal recovery in adult patients.

Relapses were common in the native kidneys of C3 p.Arg161Trp patients, occurring in 82% of children and 86% of adults, which is considerably higher than the 20% to 40% relapse risks typically reported in CaHUS cohorts.^7^^,^^10^^,^^20^^,^^26^, ^27^, ^28^ Our finding is strengthened by a similar relapse risk of 88% (*n* = 15/17) in patients with CaHUS with the C3 p.I1157T variant, a variant frequently seen in Japan and with functional consequences similar to C3 p.Arg161Trp.^13^^,^^29^ In contrast to other CaHUS cohorts, our study benefits from longer observation, thereby identifying late relapses that might not have been captured previously. In addition, almost 90% of first relapses could be classified as definite.

There appeared to be a shorter time to first relapse in adults who had received eculizumab than in adults who did not. This may reflect increased awareness for relapses, now that complement inhibition therapy is available, evidenced by the fact that all suspected relapses were observed in the post-eculizumab era. In addition, because eculizumab enables recovery in patients who formerly would have progressed to ESKD, the group of patients at risk for relapse differs from historical cohorts.

Despite frequent relapses, the long-term outcomes of C3 p.Arg161Trp patients seem generally favorable in the present era. Especially in patients with disease onset in childhood, because 9 of 11 children still had eGFR > 90 ml/min per 1.73 m^2^ at the end of follow-up. In adults, all relapses treated with eculizumab, except 1, resulted in no additional kidney function loss. These data fit with previous findings on the safety of eculizumab discontinuation.^10^^,^^26^^,^^27^^,^^30^, ^31^, ^32^

Interestingly, and somewhat unexpected, children and young adults who presented with severe hemolysis and thrombocytopenia, but relatively preserved kidney function, showed full recovery of kidney function without anything beyond supportive therapy. Although this might suggest that not all patients require eculizumab, our data do not allow firm conclusions. Likewise, we cannot exclude that the CaHUS episode induces or predisposes to chronic kidney injury, and that this may be prevented by prompt initiation of eculizumab. These data may offer new perspectives on the broader discussion on when eculizumab is indicated and should inform future research.

This study has limitations associated with the nature of the retrospective design. Including patients from both pre- and post-eculizumab eras provided a large cohort of patients; however, the inherent shift in clinical management and monitoring over time made associations between therapy and outcomes hard to assess. In addition, there is a risk for selection bias on severity, because only patients who received care or consultation at a university medical center were included. This could have resulted in missing milder or subclinical disease episodes, as well as missing patients presenting with chronic kidney disease or ESKD *e causa ignota*. However, in a Dutch study of 340 people with unexplained chronic kidney disease, genetic testing did not reveal the C3 p.Arg161Trp variant.^33^ Another limitation is the missing data of some CaHUS episodes, which occasionally led to small sample sizes, especially in subgroup analyses, making it more difficult to draw firm conclusions. Lastly, eGFR was calculated using different formulas for adults and children, complicating the direct statistical comparison between these groups.

In summary, this study shows significant differences in the presentation and outcomes of children and adults with CaHUS. Children presented with milder AKI, but had more profound hemolysis. The latter may serve as a potential hallmark of C3 p.Arg161Trp. In addition, the C3 p.Arg161Trp is associated with a high risk of relapse in native kidneys (> 80%). Despite frequent relapses, the prognosis of C3 p.Arg161Trp appears favorable, particularly for patients with disease onset in childhood.

## Disclosure

NCAvdK received consultancy fees from Samsung and Novartis. CD received a consultancy fee from Sobi. JFMW received consultancy fees from Novartis, Otsuka, and ClimbBio. All the other authors declared no competing interests.

## Supplementary Material

Supplementary File (PDF) **Method S1. Definition of CKD and hypertension. Method S2. CaHUS episodes classification. Results S1. Details on patient with gradual loss of kidney function before CaHUS presentation. Supplementary References. Figure S1.** Localization and functional characterization of C3 p.Arg161Trp. Figure S2. Ten-year end-stage kidney disease-free survival of children and adults with the C3 p.Arg161Trp variant. Figure S3. Changes in eGFR during follow-up of 10 C3 p.Arg161Trp adults with ESKD-free survival. Table S1. Analysis of additional variants in CaHUS-associated genes in C3 p.Arg161Trp patients. **Table S2.** Symptoms at CaHUS presentation of C3 p.Arg161Trp patients. **Table S3.** Kidney function recovery outcomes after onset of C3 p.Arg161Trp adult patients stratified by presentation in pre- or post-eculizumab era. STROBE Checklist.
