# Supplementary material for: Atypical Hemolytic Uremic Syndrome in Children and Adults With the Hot Spot C3 Gene Variant p.Arg161Trp
Source: Kidney Int Rep. 2026 May 16;11(8):106599. doi: 10.1016/j.ekir.2026.106599 (PMC13273819; doi:10.1016/j.ekir.2026.106599)
Supplement: Supplementary File (PDF) — Method S1. Definition of CKD and hypertension. Method S2. CaHUS episodes classification. Results S1. Details on patient with gradual loss of kidney function before CaHUS presentation. Supplementary References. Figure S1. Localization and functional characterization of C3 p.Arg161Trp. Figure S2. Ten-year end-stage kidney disease-free survival of children and adults with the C3 p.Arg161Trp variant. Figure S3. Changes in eGFR during follow-up of 10 C3 p.Arg161Trp adults with ESKD-free survival. Table S1. Analysis of additional variants in CaHUS-associated genes in C3 p.Arg161Trp patients. Table S2. Symptoms at CaHUS presentation of C3 p.Arg161Trp patients. Table S3. Kidney function recovery outcomes after onset of C3 p.Arg161Trp adult patients stratified by presentation in pre- or post-eculizumab era. STROBE Checklist. [file mmc1.pdf]

SUPPLEMENTARY MATERIAL

Table of contents

Supplementary Methods

Supplementary Method S1. Definition of CKD and hypertension

Supplementary Method S2. CaHUS episodes classification

Supplementary Results

Supplementary Results S1. Details on patient with gradual loss of kidney function before CaHUS presentation

Supplementary Table S1. Analysis of additional variants in CaHUS-associated genes in C3 p.Arg161Trp patients

Supplementary Table S2. Symptoms at CaHUS presentation of C3 p.Arg161Trp patients

Supplementary Table S3. Kidney function recovery outcomes after onset of C3 p.Arg161Trp adult patients stratified by presentation in pre- or post-eculizumab era

Supplementary Figure S1. Localization and functional characterization of C3 p.Arg161Trp

Supplementary Figure S2. Ten-year end-stage kidney disease-free survival of children and adults with the C3 p.Arg161Trp variant

Supplementary Figure S3. Changes in eGFR during follow-up of ten C3 p.Arg161Trp adults with ESKD-free survival

Supplementary References

STROBE Checklist

Supplementary Method S1 – Definition of CKD and hypertension

Chronic kidney disease (CKD) was defined according to the KDIGO guideline.[S1]

Hypertension was defined as a systolic blood pressure ≥130 mmHg and/or diastolic blood pressure ≥80 mmHg in adults, or ≥90th percentile in children, based on age- and sex-based pediatric

reference charts.[S2] Severe hypertension was defined as a systolic blood pressure ≥180 mmHg and/or diastolic blood pressure ≥120 mmHg in adults, ≥95th percentile +12 mmHg in children, or presence of hypertension with target-organ damage (besides AKI). In cases where blood pressure data were missing, a treating physician's diagnosis of (severe) hypertension was accepted.

Supplementary Method 2 – CaHUS episodes classification

Laboratory values and histology of patients were evaluated for classification of first episodes and relapses. The following definitions were used:

1) Laboratory evidence of TMA: ≥2 of the following criteria: thrombocytopenia (platelet count <150 x 10^9/l), lactate dehydrogenase (LDH) above the upper limit of normal (>250 U/l), and low/undetectable haptoglobin (<0.3 mg/l).
2) Histological evidence of acute/active TMA: presence of glomerular capillary, arterial and/or arteriolar thrombosis.
3) AKI: increase in serum creatinine of ≥26.53 μmol/l (0.3 mg/dl) in 48 hours or ≥1.5 times baseline in <7 days.
4) AKD: increase in serum creatinine of ≥1.5 times in 8 days to 3 months.

Classification of first episodes:

First CaHUS episodes were retrospectively analyzed to characterize presentation of CaHUS. We determined presence of AKI and laboratory and/or histological evidence of acute TMA. If baseline serum creatinine for adult patients was unknown, baseline was defined as the age-related upper limit of normal. If baseline serum creatinine for pediatric patients was unknown, baseline was defined as serum creatinine corresponding to an eGFR of 120 ml/min/1.73 m^2.

Supplementary Results S1 – Details on patient with gradual loss of kidney function before CaHUS presentation

This patient was previously screened as a kidney donor for her daughter with CaHUS-related ESKD. At that time (nine years before CaHUS onset), she had microalbuminuria of 68 mg/day and an eGFR of 74 ml/min/1.73 m^2 (CKD stage G2A2). She also suffered from pre-existent hypertension and familial hypercholesterolemia. She was deemed unfit for kidney donation. Medical records stated that her kidney function slowly deteriorated over the years; two years before CaHUS onset she had an eGFR of 39 ml/min/1.73 m^2. She was presumed to suffer from hypertensive nephropathy.

This patient was 62 years old at CaHUS onset. She presented with acute kidney injury (serum creatinine 254 µmol/l), TMA (thrombocytes 117x10^9/l, LDH 303 U/l, haptoglobin unknown), and poorly controlled hypertension. She was initially diagnosed with hypertension-induced TMA for which antihypertensive treatment was intensified. She presented again less than a month later with worsening of TMA (thrombocytes 100x10^9/l, LDH 429 U/l, haptoglobin <0.10 g/l) and kidney function (serum creatinine 278 µmol/l) despite adequate blood pressure control. Based on a clinical suspicion of CaHUS, plasma therapy was started. She showed initial recovery of TMA upon start of plasma therapy but kidney function did not improve. Kidney biopsy showed signs of mainly chronic TMA. Eculizumab was initiated 61 days after TMA presentation. Kidney function did not recover and eculizumab was discontinued after 6.4 months. One and a half months after discontinuation, patient started with renal replacement therapy.

Supplementary Table S1 – Analysis of additional variants in CaHUS-associated genes in C3 p.Arg161Trp patients

Patient nr.[a]	Age group	MCP*ggaac* haplotype homozygosity[b]	CFH haplotype homozygosity[c]	CFH auto antibodies	Other identified complement genetic variants						Missing
					Gene	cDNA level	Prot level	Heterozygous/ homozygous	MAF (gnomAd v4.1.0)	Classification (class)	
1	Adult	All but c.-652G	Negative (heterozygosity)	Negative							CFHR1-5, DGKE, MLPA for CFH/CFHR4
2	Child	Positive	Negative	Negative	*CFI*<br>*DGKE*	c.392T>G<br>c.465-3C>T	p.Leu131Arg<br>-	Heterozygous<br>Heterozygous	0.00031%<br>0.041%	Likely pathogenic (IV)[S3]<br>Uncertain significance (III)	
3	Adult	Positive	Negative (heterozygosity)	Negative	*CFB*	c.26T>A	p.Leu9His	Heterozygous	4.2%	Likely benign (II)[S4]	
4	Adult	Positive	Negative (heterozygosity)	Negative							
5	Adult	Positive	Only c.-331C>T (others heterozygous)	Negative							CFH, CFI, CFB, MCP, CFHR1-5, DGKE, MLPA for CFH/CFHR4
6	Adult	Negative (heterozygosity)	Negative (heterozygosity)	Negative							
7	Child	Positive	Negative	Negative							DGKE
8	Adult	Negative (heterozygosity)	All but c.-331C>T (heterozygous)	Negative							CFHR1-5, DGKE, MLPA for CFH/CFHR4
9	Child	Positive	Negative	Negative							
10	Adult	Negative	Negative (heterozygosity)	Negative							CFHR1-5, DGKE, MLPA for CFH/CFHR4
11	Child	Positive	Negative	Negative	*CFB*	c.1697A>C	p.Glu566Ala	Heterozygous	1.0%	Likely benign (II)	
12	Adult	Negative	Positive	Unknown							CFHR1-5, DGKE, MLPA for CFH/CFHR4
13	Adult	Positive	Negative (heterozygosity)	Negative							CFHR1-5, DGKE, MLPA for CFH/CFHR4
14	Child	Positive	Negative (heterozygosity)	Unknown							
15	Child	Negative (heterozygosity)	Positive	Negative							
16	Adult	Negative	Negative	Unknown							CFHR1-5, DGKE, MLPA for CFH/CFHR4
17	Adult	All but c.-652G	Negative (heterozygosity)	Unknown							CFHR1-5, DGKE, MLPA for CFH/CFHR4
18	Adult	Positive	Negative	Unknown							CFHR1-5, DGKE, MLPA for CFH/CFHR4

19	Adult	Positive	Positive	Negative							CFHR1-5, DGKE, MLPA for CFH/CFHR4
20	Adult	Negative	Negative	Unknown							CFHR1-5, DGKE, MLPA for CFH/CFHR4
21	Adult	All but c.-652G	Negative	Unknown							CFHR1-5, DGKE, MLPA for CFH/CFHR4
22	Adult	Positive	Negative (heterozygosity)	Unknown							CFHR1-5, DGKE, MLPA for CFH/CFHR4
23	Child	Positive	Negative	Unknown							
24	Adult	Positive	Negative	Negative							CFHR1-5, DGKE
25	Child	Positive	Negative	Negative							
26	Child	Positive	Negative (heterozygosity)	Unknown							
27	Adult	Positive	Negative (heterozygosity)	Unknown							CFHR1-5, DGKE
28	Adult	Positive	Negative (heterozygosity)	Unknown							CFH, CFI, MCP, DGKE
29	Adult	Positive	Negative (heterozygosity)	Unknown							CFHR1-5, DGKE
30	Adult	Negative (heterozygosity)	Negative	Unknown							
31	Adult	Positive	Negative	Unknown	*CFH*	c.1204C>T	p.His402Tyr	Heterozygous	63.7%	Likely benign (II)[S5]	CFHR1-5, DGKE, MLPA for CFH/CFHR4
32	Adult	Unknown	Negative (heterozygosity)	Unknown	*C3*	c.3773A>C	p.Glu1258Ala	Heterozygous	Not present	Uncertain significance (III)	CFHR1-5, DGKE
33	Child	Negative	Negative (heterozygosity)	Unknown							CFB, CFHR1-5, DGKE, MLPA for CFH/CFHR4
34	Adult	Negative	Negative	Negative							
35	Child	Positive	Negative (heterozygosity)	Negative							
36	Adult	Negative	Negative	Unknown							
37	Adult	Positive	Negative (heterozygosity)	Negative	*C3*	c.463A>C	p.Lys155Gln	Heterozygous	0.38%	Uncertain significance (III)[S6]	

Abbrevations: C3, complement protein 3; CFB, complement factor B; CFH, complement factor H; CFHR, complement factor H-related; CFI, complement factor I; DGKE, diacylglycerol kinase epsilon; DNA; Deoxyribonucleic Acid; MAF, Minor Allele Frequency; MCP, membrane cofactor protein; MLPA, Multiplex Ligation-Dependent Probe Amplification.

[a] Patients 1, 2, 3, 4, and 24 correspond to patients 11, 5, 12, 16, 18 in Bouwmeester et al.[10] Patients 8 and 14 correspond to patients 4 and 11 in Duineveld et al.[18]

[b] The risk haplotype MCP*ggaac* is formed by: c.−652A>G (rs2796267), c.−366A>G (rs2796268), c.IVS9−78G>A (rs1962149), c.IVS12+638G>A (rs859705), and c.4070T>C (rs7144).

[c] The risk haplotype CFH-H3 is formed by: c.-331C>T (rs3753394), c.2016A>G (rs3753396), and c.2808G>T (rs1065489).

Supplementary Table S2 – Symptoms at CaHUS presentation of C3 p.Arg161Trp patients

		Total[a] N = 37		Children (C) *n* = 11		Adults (A) *n* = 26	*P* value C vs. A
**Symptoms at presentation**							
**No symptoms**	n=31	0	n=10	0	n=21	0	
**Fever**	n=31	14 (45%)	n=10	7 (70%)	n=21	7 (33%)	*P* = 0.121
**Purpura**	n=31	1 (3%)	n=10	0	n=21	1 (5%)	*P* = 1.000
**Petechiae**	n=31	6 (19%)	n=10	4 (40%)	n=21	2 (10%)	*P* = 0.067
**Hematoma**	n=31	3 (10%)	n=10	2 (20%)	n=21	1 (5%)	*P* = 0.237
**Nausea/vomiting**	n=31	14 (45%)	n=10	6 (60%)	n=21	8 (38%)	*P* = 0.441
**Abdominal pain**	n=31	8 (26%)	n=10	4 (40%)	n=21	4 (19%)	*P* = 0.381
**Diarrhea**	n=31	6 (19%)	n=10	4 (40%)	n=21	2 (10%)	*P* = 0.067
**Jaundice**	n=31	7 (23%)	n=10	5 (50%)	n=21	2 (10%)	***P* = 0.022**
**Edema**	n=31	9 (29%)	n=10	1 (10%)	n=21	8 (38%)	*P* = 0.205
**Red-colored urine**	n=31	14 (45%)	n=10	9 (90%)	n=21	5 (24%)	***P* = 0.001**
**Dyspnea**	n=31	6 (19%)	n=10	0	n=21	6 (29%)	*P* = 0.141
**Coughing**	n=31	7 (23%)	n=10	2 (20%)	n=21	5 (24%)	*P* = 1.000
**Headache**	n=31	9 (29%)	n=10	1 (10%)	n=21	8 (38%)	*P* = 0.205
**Blurred vision**	n=31	2 (6%)	n=10	0	n=21	2 (10%)	*P* = 1.000
**Confusion**	n=31	2 (6%)	n=10	0	n=21	2 (10%)	*P* = 1.000
**Oliguria/anuria**	n=31	1 (3%)	n=10	1 (10%)	n=21	0	*P* = 0.323

[a] n refers to the number of patients for whom data were available.

Supplementary Table S3 – Kidney function recovery outcomes after onset of C3 p.Arg161Trp adult patients stratified by presentation in pre- or post-eculizumab era

	Treatment	Full or partial recovery	ESKD	Total
**Pre-eculizumab era**	Supportive treatment or PE	6 (43%)	8 (57%)	14
**Post-eculizumab era**	Supportive treatment or PE	2 (50%)	2 (50%)	4
	Eculizumab ± PE	7 (88%)	1 (13%)	8

Percentages may not total 100% due to rounding.
Abbrevations: ESKD, end-stage kidney disease; PE, plasma exchange.

Supplementary Figure S1 – Localization and functional characterization of C3 p.Arg161Trp

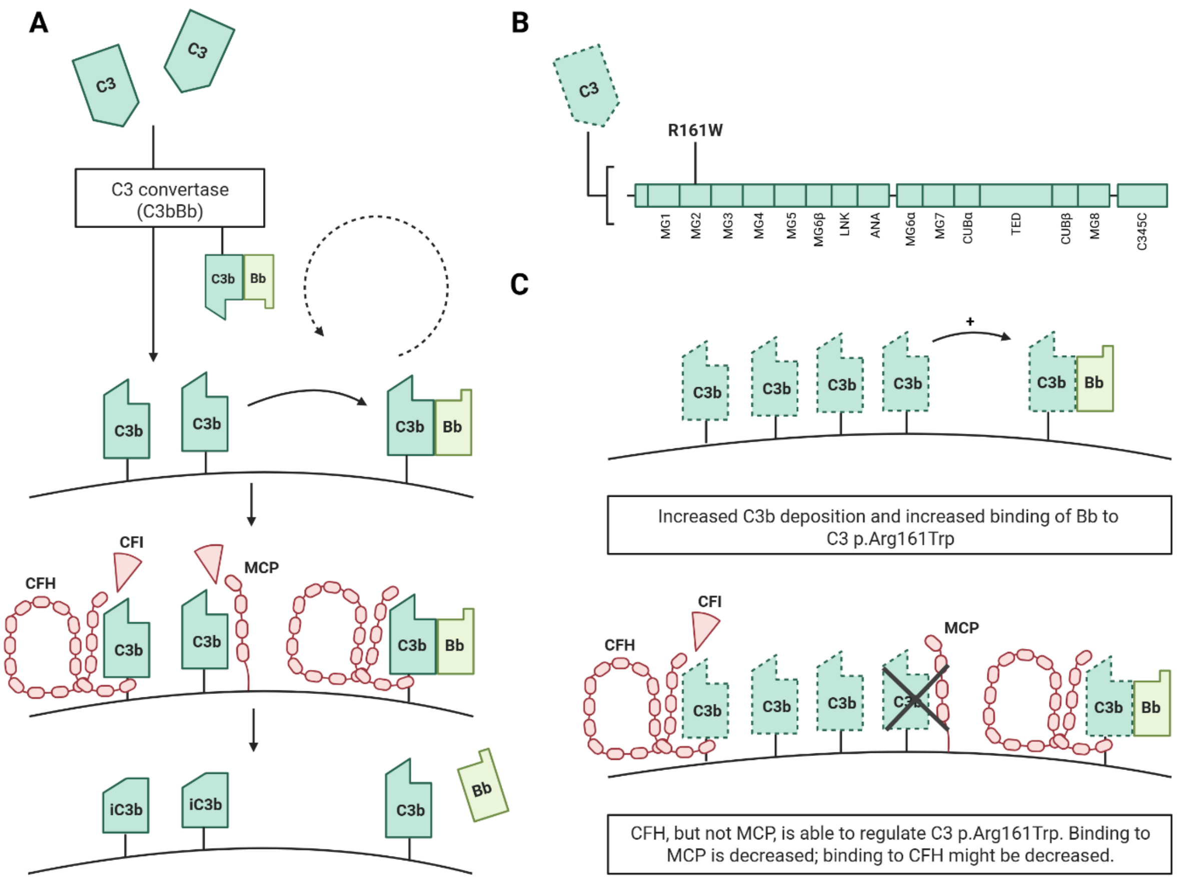

A) Overview of the activation and regulation of the alternative pathway. Central component C3 is cleaved by C3 convertases, such as the alternative pathway C3 convertase C3bBb, producing C3b. The binding of factor B (cleaved to Bb by factor D) to C3b promotes the formation of more C3 convertases, creating an amplification loop, both in fluid phase and on surfaces. To prevent excessive activation and host tissue injury, the alternative complement pathway is controlled by regulatory proteins like complement factor H (CFH), complement factor I (CFI), and membrane cofactor protein (MCP). CFH and MCP serve as cofactors for CFI in degrading C3b into iC3b. In addition, CFH facilitates the disassembly of the C3 convertase by displacing factor Bb. **B)** The C3 protein consists of 13 domains. The C3 p.Arg161Trp variant is localized in the macroglobulin 2 (MG2) domain. Minor allele frequency of C3 p.Arg161Trp in a European (non-Finnish) reference population from gnomAD v4.1.0. was 0.00042%.[S7] **C)** C3 p.Arg161Trp is both a direct and indirect gain of function variant. C3 p.Arg161Trp is resistant to regulation by MCP and causes weakening of the binding with MCP.[12,13] The variant has increased binding to Bb and is associated with increased C3b deposition.[12,13] The binding to CFH might be decreased; binding of C3 p.Arg161Trp to CFH was significantly decreased in an enzyme-linked immune sorbent assay (ELISA) with high concentration range of C3b (up to 1000 ng/mL) but not with surface plasmon resonance (SPR) or in an ELISA with lower concentration range (up to 300 ng/mL).[12,13,S8] Figure created with Biorender.

Supplementary Figure S2 – Ten-year end-stage kidney disease-free survival of children and adults with the C3 p.Arg161Trp variant

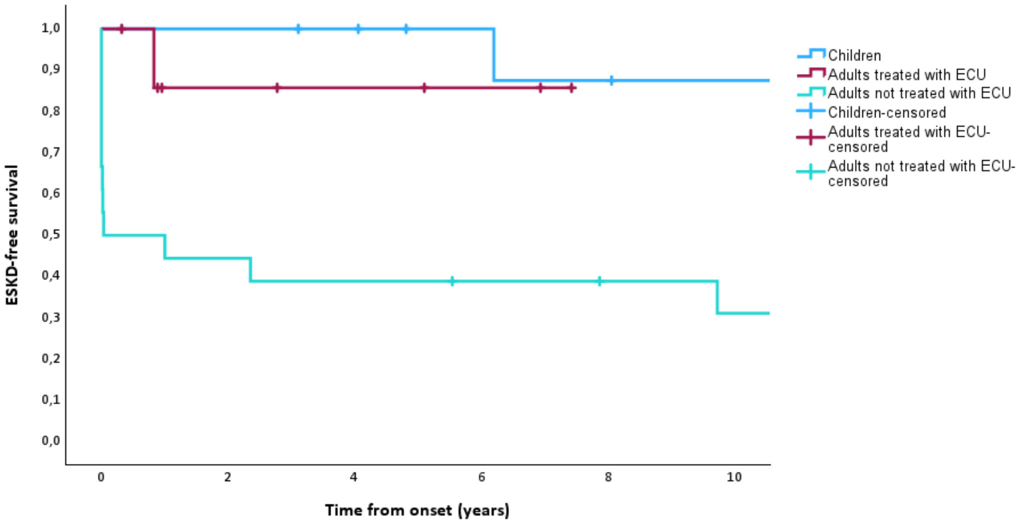

**Number at risk**						
Children	11	11	10	8	7	6
Adults treated with ECU	8	4	3	2	0	0
Adults not treated with ECU	18	8	7	6	5	4

Abbrevations: ESKD, end-stage kidney disease.

Supplementary Figure S3 – Changes in eGFR during follow-up of ten C3 p.Arg161Trp adults with ESKD-free survival

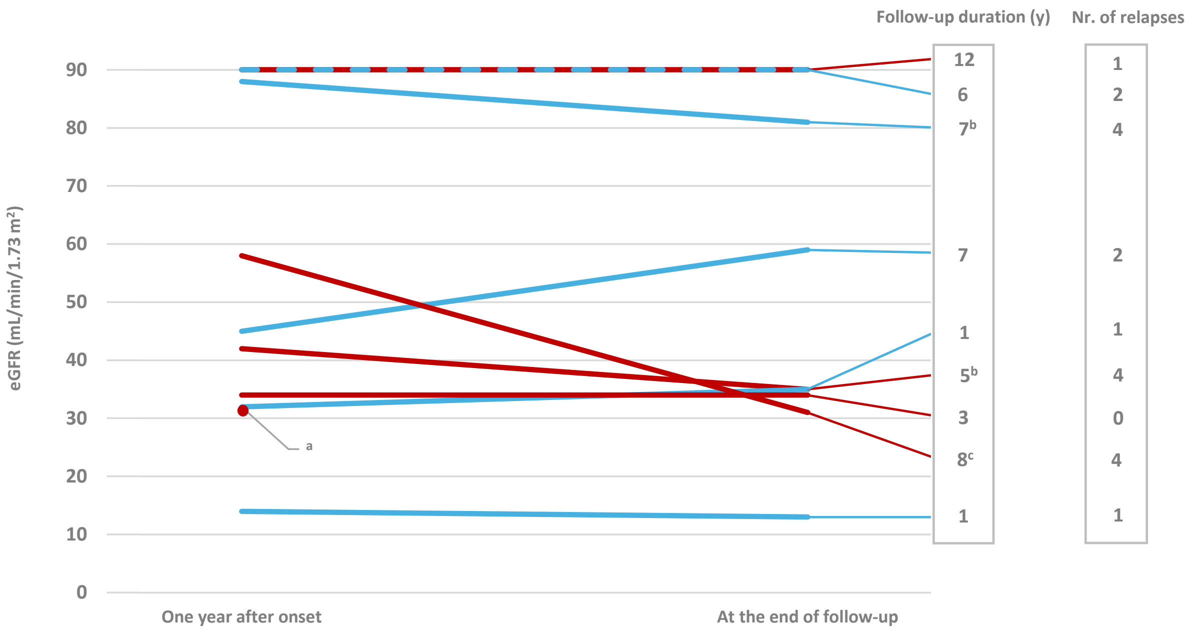

eGFR values >90 ml/min/1.73 m² are plotted as 90 ml/min/1.73 m² as the CKD-EPI equation is less reliable in this range.

Abbrevations: eGFR, estimated glomerular filtration rate; y, years.

[a] This patient had a follow-up duration of 0.3 months. The dot marks the eGFR three months after onset (32 ml/min/1.73 m^2). This patient was still on eculizumab at the end of the follow-up.

[b] In two patients, kidney function recovered to baseline after relapses (defined as a <10% increase in serum creatinine after relapse relative to baseline), yet eGFR declined during follow-up. In one patient, eGFR declined from 88 to 81 ml/min/1.73 m^2 over seven years, consistent with the expected age-related decline of approximately 1 ml/min/1.73 m^2.[S9] In the other patient, eGFR declined from 42 to 35 ml/min/1.73 m^2 over five years. One month before the end of follow-up, this patient had restarted eculizumab treatment for a fourth relapse. Since restart an ongoing improvement of kidney function is seen.

[c] At one year after onset of CaHUS, this patient's eGFR was 58 ml/min/1.73 m^2. Four years later, kidney function first slowly declined to an eGFR of 48 ml/min/1.73 m², and thereafter a more rapid progression of eGFR loss was seen. A first relapse was diagnosed (the kidney biopsy showed predominantly chronic TMA), and eculizumab treatment was started. After two dosages of eculizumab treatment was discontinued due to presentation with colitis, initially suspected to be an adverse event of eculizumab. Kidney function failed to recover to baseline and stabilized at 32 ml/min/1.73 m^2. During the next three relapses, eGFR repeatedly returned to this level.

Supplementary References

S1. Stevens PE, Ahmed SB, Carrero JJ, et al. KDIGO 2024 Clinical Practice Guideline for the Evaluation and Management of Chronic Kidney Disease. *Kidney International*. 2024;105(4):S117-S314. doi:10.1016/j.kint.2023.10.018

S2. Flynn JT, Kaelber DC, Baker-Smith CM, et al. Clinical Practice Guideline for Screening and Management of High Blood Pressure in Children and Adolescents. *Pediatrics*. Sep 2017;140(3)doi:10.1542/peds.2017-1904

S3. de Jong S, Volokhina EB, de Breuk A, et al. Effect of rare coding variants in the CFI gene on Factor I expression levels. *Hum Mol Genet*. Aug 11 2020;29(14):2313-2324. doi:10.1093/hmg/ddaa114

S4. Hecker LA, Edwards AO, Ryu E, et al. Genetic control of the alternative pathway of complement in humans and age-related macular degeneration. *Hum Mol Genet*. Jan 1 2010;19(1):209-15. doi:10.1093/hmg/ddp472

S5. Martín Merinero H, Zhang Y, Arjona E, et al. Functional characterization of 105 factor H variants associated with aHUS: lessons for variant classification. *Blood*. 2021;138(22):2185-2201. doi:10.1182/blood.2021012037

S6. Le Clech A, Simon-Tillaux N, Provôt F, et al. Atypical and secondary hemolytic uremic syndromes have a distinct presentation and no common genetic risk factors. *Kidney International*. 2019/06/01/ 2019;95(6):1443-1452. doi:https://doi.org/10.1016/j.kint.2019.01.023

S7. Chen S, Francioli LC, Goodrich JK, et al. A genomic mutational constraint map using variation in 76,156 human genomes. *Nature*. 2024/01/01 2024;625(7993):92-100. doi:10.1038/s41586-023-06045-0

S8. Volokhina E, Westra D, Xue X, Gros P, van de Kar N, van den Heuvel L. Novel C3 mutation p.Lys65Gln in aHUS affects complement factor H binding. *Pediatr Nephrol*. Sep 2012;27(9):1519-24. doi:10.1007/s00467-012-2183-z

S9. Waas T, Schulz A, Lotz J, et al. Distribution of estimated glomerular filtration rate and determinants of its age dependent loss in a German population-based study. *Scientific Reports*. 2021/05/13 2021;11(1):10165. doi:10.1038/s41598-021-89442-7

STROBE Statement—Checklist of items that should be included in reports of *cohort studies*

	Item No	Recommendation	Page No
**Title and abstract**	1	(*a*) Indicate the study's design with a commonly used term in the title or the abstract	2
		(*b*) Provide in the abstract an informative and balanced summary of what was done and what was found	2
**Introduction**			
Background/ rationale	2	Explain the scientific background and rationale for the investigation being reported	3/4
Objectives	3	State specific objectives, including any prespecified hypotheses	4
**Methods**			
Study design	4	Present key elements of study design early in the paper	4
Setting	5	Describe the setting, locations, and relevant dates, including periods of recruitment, exposure, follow-up, and data collection	4
Participants	6	(*a*) Give the eligibility criteria, and the sources and methods of selection of participants. Describe methods of follow-up	4/5/6
		(*b*) For matched studies, give matching criteria and number of exposed and unexposed	NA
Variables	7	Clearly define all outcomes, exposures, predictors, potential confounders, and effect modifiers. Give diagnostic criteria, if applicable	5/6
Data sources/ measurement	8*	For each variable of interest, give sources of data and details of methods of assessment (measurement). Describe comparability of assessment methods if there is more than one group	5/6
Bias	9	Describe any efforts to address potential sources of bias	13
Study size	10	Explain how the study size was arrived at	See figure 1
Quantitative variables	11	Explain how quantitative variables were handled in the analyses. If applicable, describe which groupings were chosen and why	6
Statistical methods	12	(*a*) Describe all statistical methods, including those used to control for confounding	6
		(*b*) Describe any methods used to examine subgroups and interactions	4
		(*c*) Explain how missing data were addressed	Nr. of patients analysed are noted in the tables
		(*d*) If applicable, explain how loss to follow-up was addressed	NA
		(*e*) Describe any sensitivity analyses	NA
**Results**			
Participants	13*	(a) Report numbers of individuals at each stage of study—eg numbers potentially eligible, examined for eligibility, confirmed eligible, included in the study, completing follow-up, and analysed	Figure 1
		(b) Give reasons for non-participation at each stage	Figure 1
		(c) Consider use of a flow diagram	See Figure 1

Descriptive data	14*	(a) Give characteristics of study participants (eg demographic, clinical, social) and information on exposures and potential confounders<br><br>(b) Indicate number of participants with missing data for each variable of interest<br>(c) Summarise follow-up time (eg, average and total amount)	Table 1 and P7/8 Genetic characteristics in supplementary file<br>Tables<br><br>10
Outcome data	15*	Report numbers of outcome events or summary measures over time	8/9/10/11
Main results	16	(*a*) Give unadjusted estimates and, if applicable, confounder-adjusted estimates and their precision (eg, 95% confidence interval). Make clear which confounders were adjusted for and why they were included<br>(*b*) Report category boundaries when continuous variables were categorized<br>(*c*) If relevant, consider translating estimates of relative risk into absolute risk for a meaningful time period	Median (ranges) were reported for all continuous measurements<br><br>Tables, e.g. for haptoglobin<br>NA
Other analyses	17	Report other analyses done—eg analyses of subgroups and interactions, and sensitivity analyses	9, e.g. the analysis between adult groups with different outcomes
**Discussion**			
Key results	18	Summarise key results with reference to study objectives	11/12/13/14
Limitations	19	Discuss limitations of the study, taking into account sources of potential bias or imprecision. Discuss both direction and magnitude of any potential bias	13/14
Interpretation	20	Give a cautious overall interpretation of results considering objectives, limitations, multiplicity of analyses, results from similar studies, and other relevant evidence	14
Generalisability	21	Discuss the generalisability (external validity) of the study results	11-12, e.g. by comparing with other CaHUS cohorts and mutation-wide
**Other information**			
Funding	22	Give the source of funding and the role of the funders for the present study and, if applicable, for the original study on which the present article is based	14/15

*Give information separately for exposed and unexposed groups.

Note: An Explanation and Elaboration article discusses each checklist item and gives methodological background and published examples of transparent reporting. The STROBE checklist is best used in conjunction with this article (freely available on the Web sites of PLoS Medicine at http://www.plosmedicine.org/, Annals of Internal Medicine at http://www.annals.org/, and Epidemiology at http://www.epidem.com/). Information on the STROBE Initiative is available at http://www.strobe-statement.org.
